# Gender Differences in Dietary Patterns and Their Association with the Prevalence of Metabolic Syndrome among Chinese: A Cross-Sectional Study

**DOI:** 10.3390/nu8040180

**Published:** 2016-03-25

**Authors:** Shu-Hong Xu, Nan Qiao, Jian-Jun Huang, Chen-Ming Sun, Yan Cui, Shuang-Shuang Tian, Cong Wang, Xiao-Meng Liu, Hai-Xia Zhang, Hui Wang, Jie Liang, Qing Lu, Tong Wang

**Affiliations:** 1 Department of Health Statistics, School of Public Health, Shanxi Medical University, 56 Xinjiannanlu Street, Taiyuan 030001, China; xushuh_sxmu@sina.com (S.-H.X.); qiaonan_sxmu@sina.com (N.Q.); cuiyan_sxmu@sina.com (Y.C.); tianshuang_sxmu@sina.com (S.-S.T.); wangcong_sxmu@sina.com (C.W.); liuxiaomengsy@163.com (X.-M.L.); zhx.196@163.com (H.-X.Z.); annieyuner@163.com (H.W.); liangjie_sxmu@sina.com (J.L.); qlu@epi.msu.edu (Q.L.); 2 Department of Neurosurgery, General Hospital of Datong Coal Mining Group, Datong 037000, China; hjj1958@126.com; 3 Department of Urology, General Hospital of Datong Coal Mining Group, Datong 037000, China; bun005@126.com; 4 Department of Health Statistics, Taiyuan Xinghualing District Food & Drug Administration, Taiyuan 030001, China; 5 Department of Epidemiology and Biostatistics, Michigan State University, East Lansing, MI 48824, USA

**Keywords:** dietary patterns, metabolic syndrome, factor analysis, invariance, cluster analysis

## Abstract

Few studies have investigated gender differences in dietary intake. The objective of this cross-sectional study was to examine gender differences in dietary patterns and their association with the prevalence of metabolic syndrome. The food intakes of 3794 subjects enrolled by a two-stage cluster stratified sampling method were collected using a valid semi-quantitative food frequency questionnaire (FFQ). Metabolic syndrome (MetS) was defined according to the International Diabetes Federation (IDF) and its prevalence was 35.70% in the sample (37.67% in men and 24.67% in women). Dietary patterns were identified using factor analysis combined with cluster analysis and multiple group confirmatory factor analysis was used to assess the factorial invariance between gender groups. The dominating dietary pattern for men was the “balanced” dietary pattern (32.65%) and that for women was the “high-salt and energy” dietary pattern (34.42%). For men, the “animal and fried food” dietary pattern was related to higher risk of MetS (odds ratio: 1.27; 95% CI: 1.01–1.60), after adjustment for age, marital status, socioeconomic status and lifestyle factors. For women, the “high-salt and energy” dietary pattern was related to higher risk of MetS (odds ratio: 2.27; 95% CI: 1.24–4.14). We observed gender differences in dietary patterns and their association with the prevalence of MetS. For men, the “animal and fried food” dietary pattern was associated with enhancive likelihood of MetS. For women, it was the “high-salt and energy” dietary pattern.

## 1. Introduction

Metabolic syndrome (MetS) is defined as a cluster of interrelated risk factors for cardiovascular disease and type 2 diabetes mellitus (T2DM), including high blood pressure, increasing fasting glucose, low high-density lipoprotein cholesterol (HDL-C) levels, elevated triglycerides (TG) levels, and central obesity [1]. Based on a cohort study conducted in 2012, the prevalence of MetS in Japan adult population was 43.6% in men and 28.9% in women [2]. The prevalence of MetS in Korea population has increased from 31.3% in 2007 to 37% between 2010 and 2012 [3,4]. With the development of economy, the prevalence of MetS in China has also increased considerably from 13.7% between 2000 and 2001 to 27.4% between 2009 and 2010 [5,6]. According to a cross-sectional survey of Jilin province, China in 2012, the adjusted prevalence of MetS has increased to 32.86% (36.64% in men and 29.66% in women) [7]. Some studies have reported that MetS can enhance the risk of cardiovascular disease, diabetes, chronic kidney disease, stroke, and nonalcoholic fatty liver disease [8,9,10]. MetS has therefore become an important global public health challenge. 

Diet is one of the major influential factors in the development of chronic disease [11,12]. Because of the complexity of diet, in which foods and nutrients more likely act in synergy rather than a simple additive fashion [13,14], dietary patterns analyses play an important role in assessing the relations between diet and disease [15,16]. Two approaches of deriving dietary patterns have been frequently used: the cluster analysis and the exploratory factor analysis (EFA) [15,16,17,18,19,20,21,22]. According to individual variations of dietary intakes, cluster analysis can assign participants to subgroups in which dietary intakes are relatively homogeneous. Factor analysis can derive dietary patterns by identifying a smaller set of common factors from the correlation structure of different food variables. In order to get the individual levels of different dietary patterns, the factor analysis combined with the cluster analysis approach has been used in previous studies [15,17,18,21,23,24].

Many studies have examined the relationship between dietary patterns and MetS using the cluster analysis or the factor analysis [22,23,25,26,27]. Certain dietary patterns including the “Western” dietary pattern, the “sweet” dietary pattern, and the “traditional” dietary pattern were positively associated with the risk of MetS, whereas the “green water” dietary pattern was inversely associated with the MetS. The “Western” dietary pattern was characterized by high intakes of red meat, fruit, eggs, caffeine and seafood. The “sweet” dietary pattern included candy, sugar, dairy desserts and sugary beverages. The “traditional” dietary pattern consisted of high intakes of cereal products, such as grain, rice, tubers and beans. The “green water” dietary pattern was characterized by high intakes of rice, fishes, dairy, vegetables and fruits [16,22,23,27,28,29,30]. However, to our knowledge, on the relationship between dietary patterns and MetS, there are few studies that examined the heterogeneity of diet across population subgroups. Given that the heterogeneous populations (e.g., mixture of racial or gender groups) were commonly included in this sort of study, it was not appropriate to derive common dietary patterns when they were likely different between subgroups. The existence of heterogeneity of diet across subgroups might have an effect on the factor structure when dietary patterns were identified using the factor analysis [31]. In other words, the nonequivalence of factor structure across subgroups meant that the existence of heterogeneity in dietary patterns between subgroups. Therefore, the equivalence of factor structure across subgroups should be examined before deriving the final dietary patterns. Our study aimed to investigate gender differences in dietary patterns and their association with the prevalence of MetS using adequate statistical analyses including EFA and the multiple group confirmatory factor analysis.

## 2. Methods

### 2.1. Study Population

During July 2013 to December 2013, a cross-sectional study was designed to collect data from a large coalmine group which consisted of 87 coalmines and 200,000 population located in the north of Shanxi Province in China. The administrative department of the coalmine group provided us the baseline data of workers that included date of birth, gender, work type and the name of coalminer for the development of the sampling frame. Based on these data, a two-stage cluster stratified sampling design was used in this survey. In the first stage of sampling, ten coalmines were randomly selected from 87 coalmines. In the second stage, participants were selected by a stratified random sampling method based on the baseline characteristic of age, gender and work type. The sample size was calculated using the PASS software package program (version 11.0 for Windows; NCSS LLC: Kaysville, UT, USA) [32] with an expected prevalence of MS of 33.9% [33] and an allowable error of 3.3%. Considering no response or other non-conforming situations, 3794 participants were recruited. Among the sampled coalminers, 9.05% of them were excluded because of missing information or extreme values. A total of 3451 coalminers (2829 men and 523 women) were included with an age range of 20 to 65 years. The average age of men and women were 42 and 40 years, respectively.

### 2.2. Definition of MetS

According to the International Diabetes Federation (IDF) 2009, any three of the following five factors were defined as MetS: (1) elevated TG: ≥1.7 mmol/L; (2) high blood pressure: ≥130/85 mmHg; (3) T2DM or fasting plasma glucose: ≥5.6 mmol/L; (4) elevated HDL-C: <1.0 mmol/L(men) and <1.3 mmol/L (women); and (5) elevated waist circumference (WC): WC ≥85 cm and 80 cm in Chinese men and women, respectively [1,34,35].

### 2.3. Blood Sample, Anthropometrics Variables and Blood Pressure

The study had been approved by Shanxi Medical University Ethics Committee and the informed written consent was obtained from each participant. The collection of blood samples and measurement of height, body weight, waist circumference and blood pressure were processed in the morning. Blood samples were obtained from the antecubital vein of subjects who had fasted for at least 10 h by trained nurses and timely brought back to hospital with a cold chain. Concentrations of TG, HDL-C and fasting plasma glucose were measured using the SIEMENS ADVIA 1800 Automatic Biochemical analyzer (JEOL Ltd, Tokyo, Japan) in the hospital’s laboratory that met the standards of the National Reference laboratory. The duplicate measurements of height, body weight and waist circumference were conducted by trained and certified investigators using standard protocols and techniques [29,36]. Height and body weight were measured in light indoor clothing without shoes. The waist circumference was measured horizontally at the midpoint between the bottom of the rib cage and the top of the iliac crest at the end of exhalation. The accuracies of measurements for barefoot height, weight, waist circumference were 1 mm, 0.1 kg and 1 mm, respectively. Body mass index (BMI) (kg/m^2^) was calculated by dividing weight by the square of height. According to the criteria recommended by the World Health Organization, participants were classified to four categories namely underweight, normal weight, overweight and obesity. The corresponding BMI value were <18.5, 18.5–24.99, 25–29.99 and ≥30 kg/cm^2^. Participants’ blood pressure was obtained by trained and certified nurses according to the recommendations for blood pressure in human [37]. The blood pressure was measured three times using mercury sphygmomanometer with the subjects in the sitting position after 5 min of rest. Subjects were required to avoid alcohol consumption, caffeinated beverages, smoking and exercise for at least 30 min before measurement [38].

### 2.4. Assessment of General Information and Covariates

In this study, interviewers who have medical knowledge and come from Shanxi Medical University were trained to conduct the investigation. The survey was conducted by face-to-face interview within 30 min. The self-administered baseline questionnaire included questions on demographics, lifestyle and medical history factors. The following variables were used in our study: age, gender, marital status (single; married; divorced), work type (heavy physical; light physical; mental labor), educational level (bachelor degree or above; junior college and senior high school; junior high school or below), monthly income (≤4000; 4000–6000; ≥6000 RMB), smoking, alcohol consumption, medical history and family history of illness. Adults who have been smoking at least one cigarette per day during the past month were defined as current smokers [39]. Individuals who have consumed alcohol at least once per month in the past year were defined as current alcohol users [40]. The international physical activity questionnaire (IPAQ) was used to assess the physical activity level. IPAQ assessed physical activity level across a comprehensive set of components including work-related activity, transport-related activity, housework activity, leisure time, exercise time, time of sitting posture and sleeping. Physical activity level was classified as inactive, minimally active and health-enhancing physical activity [41].

### 2.5. Assessment of Dietary Intake

A validated semi-quantitative food frequency questionnaire was designed to collect information of the dietary intakes in the past year [42,43]. The FFQ included information regarding the consumption of 20 food groups. The 20 food groups were as follows: rice, wheat flour, cereal, potatoes, fried dough, pork, red meat, poultry, viscera, fish and shrimp, dairy products, beans and bean products, eggs and egg dishes, vegetables, pickled vegetables, salted and preserved vegetables, vermicelli, pastry, fruits, and nuts. Participants were asked to indicate the amount of 20 food groups they had consumed over the past year, the cycle they had consumed (daily, weekly, monthly, yearly and never) and the frequency. The unit of the average food intake per day was Liang, which is equivalent to 50 g. The cycle they had consumed was converted to the number of intakes per day. The calculation of dietary intake was obtained by computing the amount of each food group consumed as per day per person.

### 2.6. Identification of Dietary Patterns

The dietary patterns were model-based using the data of dietary intakes, which had been standardized and log-transformed to satisfy the normality assumption prior to analysis. Firstly, EFA was conducted using the method of principal components and varimax rotation in the entire population. The number of factors was decided based on eigenvalues, scree test, and factor interpretability [15,16]. Food groups with factor loadings ≥0.3 were retained in the corresponding factors [17]. Secondly, the equivalence of factor structure across gender was examined to assess the heterogeneity of diet. In order to check the equivalence of factor structure (factor numbers, factor loadings, intercept, factor variances, *etc.*), we conducted the factorial invariance test, which included configural invariance, weak invariance, strong invariance, and so on [44,45]. If configural invariance was existed across gender, this meant that common factor structure was maintained across gender. A failure of configural invariance occurred when factor structure was completely different. In other words, dietary patterns across gender were completely different. Weak invariance existed if factor loadings for each group were equivalent. The failure of weak invariance meant that the correlation between food groups and corresponding factor for each group was different. Strong invariance was existed if the model where intercept was constrained to be equal provided the best fit to the data. The possible reason for the lack of strong invariance lay in group variation in the means of the specific factors [46]. As an extension of confirmatory factor analysis, the multiple group confirmatory factor analysis was utilized to assess the invariance of estimated parameters of nested models across groups. The nested models were a series of models with different restrictions. The first model was fitted with all parameters free to vary between groups to assess the configural invariance. The second model was fitted to assess the weak invariance by constraining factor loadings to be equal across groups. The third model was fitted to assess the strong invariance by constraining the intercepts to be equal across groups [44,46]. If it was identified that the model fit was virtually worse when restrictions were added in a step, the modeling process should stop [44]. Considering the difficulty for fitting model, only food groups with loading values great than 0.2 for a given factor were included in the initial model [31]. We evaluated the goodness-of-fit of models using chi-square statistic, comparative fit index (CFI) and root mean squared error of approximation (RMSEA). RMSEA values less than 0.08 were considered to fit acceptably [47]. For all models, a decrease in CFI greater than or equal to −0.01 and an increase in RMSEA greater than or equal to 0.015 indicated non-invariance [48,49]. Finally, we used the EFA analysis combined with the cluster analysis to derive the dietary patterns. Factor scores were standardized before the cluster analysis. Hierarchical cluster analysis was utilized to decide number and the initial cluster centers of clusters [18]. Then the K-means cluster analysis was conducted to identify dietary patterns. By comparing cluster means of factor scores and the ratio *R*^2^/(1 − *R*^2^) of each cluster, we can infer which factors were most important in defining the differences among clusters [50].

### 2.7. Statistic Methods

All data were double-entered and managed by Epi info version 3.5.1 (CDC, Atlanta, GA, USA). Statistical analyses were performed using SAS 9.2 software (SAS Institute, Inc., Cary, NC, USA) and LISREL version 8.7 software (K.G. Joreskog and D. Sorbom, Scientific Software International, Inc., Chicago, IL, USA). A *p*-value < 0.05 was considered statistically significant. All independent variables including demographic factors and life styles were categorical variables (see Table S1) and described by frequency distribution among men and women separately. To explore the relationship between independent variables and MetS, the crude odds ratios (OR) and their 95% confidence intervals (95% CI) were estimated by the univariate logistic regression.

In the model building of dietary patterns, EFA and the K-means cluster analysis were performed separately by PROC FACTOR and PROC FASTCLUS in SAS. Each factor was labeled using the name of food groups that were positively related. Dietary patterns were named according to the results of cluster analysis. Distributions of sample characteristics among dietary patterns were examined using the chi-square test. The relationship between dietary patters and MetS was performed using univariate and multivariable logistic regression. The adjusted odds ratios (ORa) were calculated successively by adjusting for age and other variables. 

## 3. Results

The prevalence of MetS was 35.70% in the study sample (37.67% in men and 24.67% in women). Among the individual components of MetS, central obesity (54.97%; 60.76% in men and 22.56% in women) was the most prevalent, followed by high blood pressure (50.50%; 53.01% in men and 36.14% in women) and hypertriglyceridemia (40.57%; 43.41% in men and 24.67% in women). The prevalence of low HDL cholesterol and hyperglycemia were 25.99% (28.79% in men and 10.33% in women) and 15.27% (16.53% in men and 8.22% in women), respectively. According to the results of univariate analysis, all independent variables except monthly income were related to the prevalence of MetS (see Table 1).

### 3.1. Dietary Patterns

Four factors were derived using the entire study sample (see Table S2). Table 2 shows goodness-of-fit statistics for model 1, model 2 and model 3, which correspond to assumptions of configural invariance, weak invariance, and strong invariance, respectively. The goodness-of-fit statistics provided acceptable estimates for all models. However, model 3 was rejected because the decrease in CFI was greater than −0.01 (∆CFI = −0.152). Considering the results suggested the lack of strong invariance, the final dietary patterns should be derived separately for subgroups. 

Table 3 and Table 4 show the factor loadings derived by the EFA analyses separately in men and women, respectively. Among men, three important factors were identified, which totally explained 28.35% of the dietary intake variance. The first factor included salted and preserved vegetables, potatoes, wheat flour, beans and bean products, vermicelli, pastry and vegetables. The second factor included red meat, viscera, poultry, fish and shrimp, pork and fried dough. The third factor included fruits, dairy products, cereal, rice, egg dishes and vegetables. Among women, four factors were identified, which totally explained 37.76% of the dietary intake variance. The first factor consisted of viscera, poultry, red meat, fried dough, fish and shrimp, rice and pork. The second factor consisted of vegetables, fruits, cereal, nuts, dairy products, fish and shrimp, and poultry. The third factor consisted of salted and preserved vegetables, pickled vegetables, wheat flour, vermicelli and fried dough. The fourth factor consisted of potatoes, pastry, cereal, beans and bean products, eggs and egg dishes. 

Four primary clusters were obtained by performing the cluster analysis separately on men and women (Table 5). For men, the first cluster, namely the “animal and fried food” dietary pattern, was characterized by high scores of the second factor and low scores of other factors. Correspondingly, the “animal and fried food” dietary pattern was characterized by high intakes of meat and fried dough, combined with low consumption of other foods. The second cluster, namely the “fruit and dairy” dietary pattern, predominantly included fruits, dairy products, cereal and vegetables. The third cluster, namely the “traditional” dietary pattern, mainly consisted of vegetables, pickled vegetables, potatoes, wheat flour and beans. The fourth cluster was named the “balanced” dietary pattern because it included all factors. For women, clusters were, respectively, named “high-salt and energy”, “vegetable and fruit”, “balanced”, and “animal and fried food” dietary pattern in the same way. As a local special dietary pattern, the “high-salt and energy” dietary pattern predominantly included preserved vegetables, pickled vegetables, wheat flour, vermicelli and viscera. The “vegetable and fruit” dietary pattern primarily consisted with vegetables, fruits, nuts, dairy products, beans and poultry.

### 3.2. Dietary Patterns and Distributions of Sample Characteristics

Table 6 and Table 7 show that four major dietary patterns were obtained using the EFA analysis combined with the cluster analysis separately performed for men and women, respectively. The main dietary pattern for men was the “balanced” dietary pattern (32.65%) and that for women was the “high-salt and energy” dietary pattern (34.42%). Among men, adults with the “balanced” and “fruit and dairy” dietary patterns tended to be characterized by under 45 years old, higher monthly income, light physical or mental labor work type and more educated than those with other dietary patterns. Individuals who consumed alcohol tended to have the “animal and fried food” dietary pattern. Among women, the “balanced” and “vegetable and fruit” dietary patterns were associated with being under 45 years old, higher monthly income and more educated. The relationship between BMI and dietary patterns was observed neither in men nor women.

### 3.3. Dietary Patterns and Metabolic Syndrome

Table 8 shows the results of univariate and multivariable logistic regression analyses. The results of univariate analysis show that the “animal and fried food” dietary pattern among men and the “high-salt and energy” dietary pattern among women were significantly related to higher risk of MetS. After adjusted for age and other variables, the “animal and fried food” dietary pattern among men was still significantly related to higher risk of MetS (ORa: 1.27; 95% CI: 1.01–1.60). For women with the “high-salt and energy” dietary pattern, the adjusted odds ratios related to higher risk of MetS was 2.27 (95% CI: 1.24–4.14).

## 4. Discussion

Based on data from a large coalmine located in the north of Shanxi Province in China, four primary dietary patterns were identified for both men and women. Two dietary patterns of them were similar, namely the “balanced” and “animal and fried food” dietary pattern. Other gender specific dietary patterns were the “traditional” and “fruit and dairy” dietary patterns for men and “high-salt and energy” and “vegetable and fruit” dietary patterns for women. In our study, the “balanced” and “high-salt and energy” dietary patterns were mostly chosen, respectively, by men and women. Dietary patterns we derived were similar to those identified in previous studies except for the “high-salt and energy” diet [16,18,22,23,25,51]. 

Consistent with the results of previous studies, members who were characterized by higher monthly income and more educated tended to have the “balanced”, “fruit and dairy”, “vegetable and fruit” dietary patterns [16,52]. In this study, adults below 45 years old tended to have the “balanced”, “fruit and dairy” and “vegetable and fruit” dietary patterns. While in the NIH-AARP Diet and Health Study, it was old people who tended to have the “vegetable and fruit” dietary pattern [52]. The difference may be caused by the different age composition of the population in studies. In the NIH-AARP Diet and Health Study, the populations were Americans over the age of 50, while in our study the average age of men and women was 42 and 40 years, respectively. The relationship between alcohol consumption and the “animal and fried food” dietary pattern that was reported in other studies was observed for men in our study, but was not observed for women because few women consumed alcohol [22,53]. Among previous studies, BMI had negative association with the “healthy” dietary pattern and positive association with the “meat and potatoes” dietary pattern [16,22]. In our study, however, we have not observed the association between BMI and dietary patterns. This phenomenon may be caused by the interactions between BMI and physical activity. Higher physical activity levels were associated with a lower prevalence of overweight [54]. Individuals with the “traditional” dietary pattern in this study tended to be characterized by light or heavy physical activity.

Gender differences in dietary patterns and their association with the prevalence of MetS were observed in this study. We treated the “balanced” dietary pattern as the reference group, which was associated with low likelihood of MetS in previous studies [55]. In accordance with previous studies, the “animal and fried food” dietary pattern was associated with enhancive likelihood of MetS for men but no relationship observed for women in our study [3,16,23]. The relationship between the “animal and fried food” dietary pattern and MetS may partly be attributable to this pattern’s unhealthy constituents according to several mechanisms. Firstly, the “animal and fried food” dietary pattern has high factor loadings for red meat, poultry and fried dough, which are a major source of total fat intake, particularly saturated fat [56]. The consumption of saturated fat has been associated with plasma lipoprotein levels, obesity and higher blood pressure levels [57,58,59,60]. Secondly, meat intake is related to the deposition of iron, especially heme-iron because raw red meat contains high levels of oxymyoglobin, deoxymyoglobin, oxyhemoglobin, deoxyhemoglobin and cytochromes [61]. It has been reported that iron overload and high ferritin concentrations were positively associated with the risk of MetS [62,63]. Therefore, high iron contents of red meat might be related to higher risk of MetS [61,64]. Thirdly, previous studies have suggested that red meat intake was related to insulin resistance, which was one of underlying mechanisms for MetS [65]. In addition, the “animal and fried food” dietary pattern was related to high alcohol consumption in our study. High alcohol consumption was positively associated with the prevalence of MetS and its components [53,66], and then both the “animal and fried food” dietary pattern and alcohol consumption were associated with an increased risk of MetS.

The “high-salt and energy” dietary pattern characterized by high intake of sodium and carbohydrate was associated with enhancive likelihood of MetS among women. As an independent risk factor for MetS, high sodium diet was also significantly associated with hypertension, type 2 diabetes mellitus, low HDL and insulin resistance [67,68,69]. Some studies have reported that insulin resistance may lead to sodium retention, thereby enhanced blood pressure responses to salt intake. Therefore, individuals with the MetS were more sensitive to a high-salt diet [70,71]. Reduction in salt intake could be a particularly important component in reducing blood pressure in individuals with MetS [72]. High carbohydrate intake from wheat flour was associated with the prevalence of MetS and its components, particularly hypertriglyceridemia [73,74]. The mechanism might be that the carbohydrate intake that exceeded energy needs was related to insulin resistance [75]. As one of high glycemic index foods, vermicelli also had a potential role in the development of MetS [76,77].

The gender differences in dietary patterns and their association with the prevalence of MetS may be caused by following reasons. The first primary one is the heterogeneity in diet intake among men and women. The difference may affect the diet-related pathology of MetS between men and women. In the National Institutes of Health-American Association of Retired Persons Diet and Health Study, the differences in diet intake among men and women were also indicated [15,52,78,79]. The association between gender differences of food selection and the observed differences of the incidence of colorectal cancer was reported [52,79]. The second possible reason was that women with the “animal and fried food” dietary pattern accounted for a relatively small proportion (5.93%). The third possible reason was that women and men completed the FFQ differently, resulting in different degrees of measurement error. Then measurement error may affect assessment of food intake and definition of dietary patterns [15,80].

This study has its strengths and limitations. The strength of our study was that instead of using the EFA analysis, which was used to derive factors, the multiple confirmatory factor analysis was utilized to assess factorial invariance across gender in dietary patterns. Gender differences in dietary patterns and their association with the prevalence of MetS were observed in this study. There are two limitations in this study. Firstly, the gender distribution was unbalanced, which was caused by the fact that the proportion of men was larger than that of women among coalminers in China. When we randomly selected adults by a two-stage cluster sampling, the unbalanced proportion of men and women in the population was reflected into the sample. Secondly, the dietary intake was assessed by FFQ over the past year in this study. As time goes on, dietary intake may change and these changes may affect the relationship between diet and MetS.

## 5. Conclusions

In our study, four meaningful dietary patterns were identified for both men and women, after the assessment of factorial invariance. Gender differences in dietary patterns and their association with the prevalence of metabolic syndrome were observed in the Chinese population. For men, the “animal and fried food” dietary pattern was associated with increased risk of MetS. For women, it was the “high-salt and energy” dietary pattern. More research is needed to explore appropriate dietary recommendations separately for men and women in the future.

## Figures and Tables

**Table 1:** The descriptive statistics of demographic factors and their association with Metabolic syndrome (MetS).

Demographic Factors	MetS (*n* (%))		OR (95% CI)	*p*
	Yes (1232)	No (2219)		
Age group, *n* (%)			**1.54 (1.40-1.68)**	**<0.0001**
≥35 years	215 (17.45)	654 (29.47)		
35–45 years	426 (34.58)	810 (36.50)		
≥45 years	591 (47.97)	755 (34.02)		
Marital status, *n* (%)				**<0.0001**
Single	25 (2.03)	134 (6.04)	**1.00**	
Married	1185 (96.19)	2052 (92.47)	**3.10 (2.01–4.77)**	**<0.0001**
Divorced	33 (1.79)	22 (1.49)	**3.57 (1.80–7.11)**	**0.0003**
Educational level, *n* (%)			**1.23 (1.10–1.38)**	**0.0004**
Bachelor degree or above	125 (10.15)	291 (13.11)		
Junior college and senior high school	737 (59.82)	1366 (61.56)		
Junior high school or below	370 (30.03)	562 (25.33)		
Work type, *n* (%)			**1.12 (1.01–1.24)**	**0.032**
Heavy physical	263 (21.35)	564 (25.42)		
Light physical	679 (55.11)	1061 (47.81)		
Mental labor	290 (23.54)	594 (26.77)		
Current smoking, *n* (%)			**1.19 (1.03–1.37)**	**0.017**
No	487 (39.53)	970 (43.71)		
Yes	745 (60.47)	1249 (56.29)		
Alcohol consumption, *n* (%)			**1.46 (1.27–1.69)**	**<0.0001**
No	653 (53.00)	1382 (62.28)		
Yes	579 (47.00)	837 (37.72)		
Monthly income (RMB), *n* (%)			0.93 (0.89–1.02)	0.127
≤4000	336 (27.27)	561 (25.28)		
4000–6000	525 (42.61)	943 (42.50)		
≥6000	371 (30.11)	715 (32.22)		
Physical activity level, *n* (%)			**0.82 (0.72–0.94)**	**0.003**
Inactive	32 (2.60)	35 (1.58)		
Minimally Active	404 (32.79)	654 (29.47)		
Health-enhancing physical activity	796 (64.61)	1530 (68.95)		
Family history, *n* (%)			**1.37 (1.19–1.57)**	**<0.0001**
No	661 (53.65)	1359 (61.24)		
Yes	571 (46.35)	860 (38.76)		
BMI (kg/m^2^), *n* (%)			**2.54 (2.27–2.84)**	**<0.0001**
Underweight	9 (0.73)	60 (2.70)		
Normal range	422 (34.25)	1350 (60.84)		
Overweight	639 (51.87)	714 (32.18)		
Obese	162 (13.15)	95 (4.28)		

Numbers in bold indicate significant associations based on a *p* value cutoff of 0.05.

**Table 2:** The goodness-of-fit statistics for three models.

Model	χ^2^	*df*	CFI	RMSEA	∆CFI	∆RMSEA
Model 1	1775.487	328	0.803	0.053	-	-
Model 2	1800.360	345	0.802	0.051	−0.001	−0.002
Model 3	2955.232	394	0.651	0.065	−0.152	0.012

**Table 3:** Factors and factor loadings derived from FFQ among Chinese coal men *.

Food Groups	Factor 1: Vegetables, Potatoes and Wheat Flour	Factor 2: Meat and Fried Dough	Factor 3: Fruits, Dairy Products, Rice and Eggs
Salted and preserved vegetables	0.533	-	-
Pickled vegetables	0.507	-	-
Potatoes	0.478	-	-
Wheat Flour	0.474	-	-
Beans and bean products	0.463	-	-
Vermicelli	0.460	-	-
Pastry	0.398	-	-
Vegetables	0.397	-	0.319
Red meat	-	0.671	-
Viscera	-	0.580	-
Poultry	-	0.575	-
Fish and shrimp	-	0.556	-
Pork	-	0.449	-
Fried dough	-	0.355	-
Nuts	-	-	-
Fruits	-	-	0.531
Dairy products	-	-	0.510
Cereal	-	-	0.501
Rice	-	-	0.354
Eggs and egg dishes	-	-	0.318
Eigen value	2.555	1.751	1.363
Percentage of variances (%) explained	12.77	8.76	6.82

* The factor loadings < 0.3 were excluded for simplicity.

**Table 4:** Factors and factor loadings derived from FFQ among Chinese coal women *.

Food Groups	Factor 1: Meat and Fried Dough	Factor 2: Fruits, Vegetables, Nuts, Dairy Products	Factor 3: Salt, Vermicelli and Wheat Flour	Factor 4: Beans, Cereal, Potatoes, and Pastry
Viscera	0.590	-	0.343	-
Poultry	0.568	0.315	-	-
Red meat	0.547	-	-	-
Fried dough	0.521	-	0.344	-
Fish and shrimp	0.485	0.363	-	-
Rice	0.411	-	-	-
Pork	0.328	-	-	-
Dairy products	-	0.492	-	-
Fruits	-	0.590	-	-
Vegetables	-	0.582	-	-
Nuts	-	0.579	-	-
Wheat Flour	-	-	0.400	-
Pickled vegetables	-	-	0.705	-
Salted and preserved vegetables	-	-	0.672	-
Vermicelli	-	-	0.506	-
Potatoes	-	-	-	0.583
Beans and bean products	-	0.376	-	0.557
Cereal	-	-	-	0.547
Pastry	-	-	-	0.506
Eggs and egg dishes	-	-	-	0.316
Eigen value	2.660	2.044	1.614	1.233
Percentage of variances (%) explained	13.30	10.22	8.07	6.17

* The factor loadings <0.3 were excluded for simplicity.

**Table 5:** Cluster means of factor scores according to clusters for men and women.

Factors	Clusters				
	Cluster 1	Cluster 2	Cluster 3	Cluster 4	*p*
Men					
Factor 1	−0.48	−1.08	0.66	0.46	**<0.0001**
Factor 2	0.36	−0.31	−0.92	0.78	**<0.0001**
Factor 3	−1.36	0.68	−0.11	0.35	**<0.0001**
Women					
Factor 1	−0.89	0.13	0.83	0.75	**<0.0001**
Factor 2	−0.30	0.49	0.27	−2.23	**<0.0001**
Factor 3	0.38	−0.94	0.87	−0.82	**<0.0001**
Factor 4	0.05	−0.10	0.05	0.07	0.484

Numbers in bold indicate significant associations based on a *p* value cutoff of 0.05.

**Table 6:** Distributions of sample characteristics among men by four dietary patterns.

Demographic Factors	“Animal and Fried Food” Dietary Pattern	“Fruit and Dairy” Dietary Pattern	“Traditional” Dietary Pattern	“Balanced” Dietary Pattern	χ^2^	*p*
*n* (%)	519 (17.73)	665 (22.71)	788 (26.91)	956 (32.65)		
Age group, *n* (%)					**125.488**	**<0.0001**
≥35 years	145 (27.94)	230 (34.59)	114 (14.47)	226 (23.64)		
35–45 years	166 (31.98)	229 (34.44)	257 (32.61)	390 (40.79)		
≥45 years	208 (40.08)	206 (30.98)	417 (52.92)	340 (35.56)		
Educational level, *n* (%)					**15.156**	**0.019**
Bachelor degree or above	56 (10.79)	100 (15.04)	48 (6.09)	113 (11.82)		
Junior college and senior high school	297 (57.23)	444 (66.77)	427 (54.19)	617 (64.54)		
Junior high school or below	166 (31.98)	121 (18.20)	313 (39.72)	226 (23.64)		
Work type, *n* (%)					**37.557**	**<0.0001**
Heavy physical	148 (28.52)	174 (26.17)	249 (31.60)	242 (25.31)		
Light physical	276 (53.18)	324 (48.72)	427 (54.19)	487 (50.94)		
Mental labor	95 (18.30)	167 (25.11)	112 (14.21)	227 (23.74)		
Alcohol consumption, *n* (%)					**79.194**	**<0.0001**
No	210 (40.46)	379 (56.99)	493 (62.56)	449 (46.97)		
Yes	309 (59.54)	286 (43.01)	295 (37.44)	507 (53.03)		
Monthly income (RMB), *n* (%)					**33.699**	**<0.0001**
≤4000	140 (26.97)	158 (23.76)	206 (26.14)	190 (19.87)		
4000–6000	224 (43.16)	267 (40.15)	385 (48.86)	460 (48.12)		
≥6000	155 (29.87)	240 (36.09)	196 (25.00)	306 (32.01)		
BMI (kg/m^2^), *n* (%)					13.573	0.138
Underweight	8 (1.54)	12 (1.80)	17 (2.16)	12 (1.26)		
Normal range	252 (48.55)	328 (49.32)	405 (51.40)	459 (48.01)		
Overweight	205 (39.50)	274 (41.20)	321 (40.74)	401 (41.95)		
Obese	54 (10.40)	51 (7.67)	45 (5.71)	84 (8.79)		

Data are *n* (%). Numbers in bold indicate significant associations based on a *p* value cutoff of 0.05.

**Table 7:** Distributions of sample characteristics among women by four dietary patterns.

Demographic Factors	“Animal and Fried Food” Dietary Pattern	“High-Salt and Energy” Dietary Pattern	“Vegetable and Fruit” Dietary Pattern	“Balanced” Dietary Pattern	χ^2^	*p*
*n* (%)	31 (5.93)	180 (34.42)	174 (33.27)	138 (26.39)		
Age group, *n* (%)					**24.548**	**0.0004**
≥35 years	11 (35.48)	35 (19.44)	57 (32.76)	51 (36.96)		
35–45 years	11 (35.48)	63 (35.00)	74 (42.53)	46 (33.33)		
≥45 years	9 (29.03)	82 (45.56)	43 (24.71)	41 (29.71)		
Educational level, *n* (%)					**34.278**	**<0.0001**
Bachelor degree or above	6 (19.35)	14 (14.14)	43 (24.71)	36 (26.09)		
Junior college and senior high school	14 (45.16)	116 (64.44)	107 (61.49)	81 (58.70)		
Junior high school or below	11 (35.48)	50 (27.78)	24 (13.79)	21 (15.22)		
Work type, *n* (%)					-	0.082 *
Heavy physical	1 (3.23)	7 (3.89)	2 (1.15)	4 (2.90)		
Light physical	17 (54.84)	88 (48.89)	67 (38.51)	54 (39.13)		
Mental labor	13 (41.94)	85 (47.22)	105 (60.34)	80 (57.97)		
Monthly income (RMB), *n* (%)					**16.771**	**0.010**
≤4000	15 (48.39)	80 (44.44)	55 (31.61)	53 (38.41)		
4000–6000	7 (22.58)	53 (29.44)	39 (22.41)	33 (23.91)		
≥6000	9 (29.03)	47 (26.11)	80 (45.98)	52 (37.68)		
Alcohol consumption, *n* (%)					-	0.450 *
No	31 (100.00)	174 (96.67)	169 (97.13)	130 (94.20)		
Yes	0 (0.00)	6 (3.33)	5 (2.87)	8 (5.80)		
BMI (kg/m^2^), *n* (%)					7.352	0.601
Underweight	1 (3.23)	6 (3.33)	7 (4.02)	6 (4.35)		
Normal range	19 (61.29)	106 (58.89)	119 (68.39)	84 (60.87)		
Overweight	8 (25.81)	58 (32.22)	43 (24.71)	43 (31.16)		
Obese	3 (9.68)	10 (5.56)	5 (2.87)	5 (3.62)		

Numbers in bold indicate significant associations based on a *p* value cutoff of 0.05. * Fisher’s exact probability.

**Table 8:** Odds ratios (95% confidence intervals) of MetS according to dietary patterns ^1^ for men and women.

Prevalence of Metabolic Syndrome	Men					Women				
	“Balanced” Dietary Pattern	“Fruit and Dairy” Dietary Pattern	“Traditional” Dietary Pattern	“Animal and Fried Food” Dietary Pattern	*p*	“Balanced” Dietary Pattern	“Vegetable and Fruit” Dietary Pattern	“High-Salt and Energy” Dietary Pattern	“Animal and Fried Food” Dietary Pattern	*p*
**Case, *n* (%)**	350 (36.61)	258 (38.80)	277 (35.15)	218 (42.00)		23 (16.67)	35 (20.11)	64 (35.56)	7 (22.58)	
**Crude OR**	1.0	1.10 (0.90, 1.35) *p* = 0.371	0.94 (0.77, 1.14) *p* = 0.528	1.25 (1.00, 1.56) ***p* = 0.042**	0.069	1.0	1.26 (0.70, 2.25) *p* = 0.437	2.76 (1.61, 4.74) ***p* = 0.0002**	1.46 (0.56, 3.78) *p* = 0.438	**0.0005**
**Age adjusted**	1.0	1.17 (0.95, 1.44) *p* = 0.141	0.85 (0.69, 1.03) *p* = 0.099	1.26 (1.01, 1.57) ***p* = 0.042**	**0.003**	1.0	1.31 (0.72, 2.38) *p* = 0.380	2.29 (1.31, 4.02) ***p* = 0.004**	1.49 (0.56, 3.99) *p* = 0.428	**0.019**
**Multivariable adjusted ^2^**	1.0	1.28 (1.03, 1.59) ***p* = 0.028**	0.98 (0.80, 1.22) *p* = 0.882	1.27 (1.01, 1.60) ***p* = 0.043**	**0.029**	1.0	1.45 (0.77, 2.73) *p* = 0.250	2.27 (1.24, 4.14) ***p* = 0.008**	1.18 (0.40, 3.52) *p* = 0.763	**0.047**

Numbers in bold indicate significant associations based on a *p* value cutoff of 0.05. ^1^ OR and 95% confidence intervals are estimated by using the “balanced” dietary pattern as a reference group. ^2^ Adjusted for age (≥35, 35–45, ≥45), grade (inactive, minimally active, health-enhancing physical activity), alcohol consumption (yes/no), marital status (single, married, divorced, widowed), family history (yes/no), educational level (bachelor degree or above, junior college and senior high school, junior high school or below), work type (heavy physical and light physical, mental labor) and BMI (underweight, normal weight, overweight and obesity).

## Supplementary Materials

The following are available online at http://www.mdpi.com/2072-6643/8/4/180/s1, Table S1: Variable Assignment Table, Table S2, Factor loadings derived from FFQ among the entire study population.

## Abbreviations

FFQ	Food frequency questionnaire
MetS	Metabolic syndrome
IDF	The International Diabetes Federation
EFA	Exploratory factor analysis
BMI	Body mass index
IPAQ	The international physical activity questionnaire
CFI	Comparative fit index
RMSEA	Root mean squared error of approximation
