# Supplementary material for: Gender Differences in Dietary Patterns and Their Association with the Prevalence of Metabolic Syndrome among Chinese: A Cross-Sectional Study
Source: Nutrients. 2016 Mar 25;8(4):180. doi: 10.3390/nu8040180 (PMC4848649; doi:10.3390/nu8040180)
Supplement: Supplementary file 1 [file nutrients-08-00180-s001.doc]

**Supplementary Materials:** **Gender Differences in Dietary Patterns and Their Association with the Prevalence of Metabolic Syndrome among Chinese:
A Cross-Sectional Study**

**Shu-Hong Xu, Nan Qiao, Jian-Jun Huang,** **Chen-Ming Sun, Yan Cui, Shuang-Shuang Tian,
Cong Wang, Xiao-Meng Liu, Hai-Xia Zhang, Hui Wang, Jie Liang, Qing Lu and Tong Wang**

**Table S1. Variable Assignment Table.**

| **Variable** | **Assignment** |
| --- | --- |
| MetS | no = 0, yes = 1 |
| Age group | ≤35 years = 1, 35–45 years = 2, ≥45 years = 3 |
| Marital Status * | single (x1 = 0, x2 = 0), married (x1 = 0, x2 = 1), divorced (x1 = 1, x2 = 0) |
| Educational Level | bachelor degree or above = 1, junior college and senior high school = 2, junior high school or below = 3 |
| Work Type | heavy physical = 1, light physical = 2, mental labor = 3 |
| Current smoking | no = 0, yes = 1 |
| Alcohol consumption | no = 0, yes = 1 |
| Monthly Income | ≤4000 yuan = 1, 4000–6000 yuan = 2, ≥6000 yuan = 3 |
| Physical activity level | Inactive = 1, minimally active = 2, health-enhancing physical activity = 3 |
| Family history | no = 0, yes = 1 |
| Body mass index (BMI) | underweight = 1, normal range = 2, overweight = 3, obesity = 4 |

* Unordered categorical variable which was analyzed using dummy variable(x1, x2) in logistic regression. Body mass index (BMI) (kg/m^2^) was calculated by dividing weight by the square of height. BMI was classified to four categories namely underweight, normal weight, overweight and obesity. The corresponding BMI value were <18.5, 18.5–24.99, 25–29.99 and ≥30 kg/cm^2^.

**Table S2.** Factor loadings derived from FFQ among the entire study population *.

| **Food Groups** | **Factor 1: Meat and Fried Dough** | **Factor 2: Beans, Wheat Flour, Potatoes** | **Factor 3: Fruits, Cereal, Rice, Dairy Products** | **Factor 4: Salt and Pickled Vegetables** |
| --- | --- | --- | --- | --- |
| Red meat | 0.664 | - | - | - |
| Viscera | 0.613 | - | - | - |
| Poultry | 0.601 | - | - | - |
| Fish and shrimp | 0.521 | - | 0.314 | - |
| Pork | 0.486 | - | - | - |
| Fried dough | 0.405 | - | - | - |
| Beans and bean products | - | 0.591 | - | - |
| Wheat Flour | - | 0.544 | - | - |
| Potatoes | - | 0.503 | - | - |
| Vegetables | - | 0.498 | - | - |
| Pastry | - | 0.449 | - | - |
| Vermicelli | - | 0.380 | - | - |
| Fruits | - | - | 0.636 | - |
| Dairy products | - | - | 0.525 | - |
| Cereal | - | - | 0.439 | - |
| Rice | - | - | 0.408 | - |
| Nuts | - | - | 0.369 | - |
| Eggs and egg dishes | - | - | - | - |
| Salted and preserved vegetables | - | - | - | 0.830 |
| Pickled vegetables | - | - | - | 0.821 |
| Eigen value | 2.576 | 1.779 | 1.408 | 1.309 |
| Percentage of variances (%) explained | 12.88 | 8.90 | 7.04 | 6.54 |

* The factor loadings < 0.3 were excluded for simplicity.
